# Environment-Friendly Electrochemical Processes

**DOI:** 10.3390/ma14061548

**Published:** 2021-03-22

**Authors:** Carlos A. Martínez-Huitle

**Affiliations:** Institute of Chemistry, Federal University of Rio Grande do Norte, Lagoa Nova, Natal, RN CEP 59078-970, Brazil; carlosmh@quimica.ufrn.br

The present water crisis is probable to grow worse in the coming decades, and this has motivated the scientific community to identify innovative, safe, and robust water treatment technologies at a lower cost and with less energy, diminishing the use of chemicals and impact on the environment [1]. Generally, conventional water treatment approaches can solve this problem satisfactorily; however, these can be sometimes chemically, energetically, and operationally intensive. Consequently, science and technology have stimulated the development of other techno–eco alternatives. 

In this frame, electrochemical processes have emerged as environment-friendly methods and sustainable technologies that offer a different solution to many environmental problems because they are versatile, efficient, cost-effective, easily automatable, and the electrons are a clean reagent (inexpensive and suitable reagent to drive the decontamination), avoiding conventional chemical oxidizers or reducing agents [2].

Over the last two decades, significant efforts have been made by several research groups around the world in order to preferably develop electrochemical-based technologies for use in conventional water and wastewater treatment methods. The recent advances in this interdisciplinary area have encouraged close collaborations between chemists, electrochemists, engineers, and other scientists, particularly in the use of these alternative technologies for monitoring and removing pollutants from wastewaters and other water matrices. Electrochemical wastewater treatments have a significant technical impact because they can be easily scaled up or designed as small–portable devices, while the development of electrochemical monitoring approaches benefits the use of these strategies in real time. 

This special issue was proposed to cover the current development status of these technologies, in removing and monitoring pollutants, emphasizing the fundamentals and new approaches developed ranging from their design, fabrication, and material properties to the potential applications. Papers focusing on electrocatalytic materials, the key component of the electrochemical cell, have been considered. The original applicability of a hybrid electrochemical sensor composed of green materials such as cork and graphite for detecting caffeine in aqueous solutions was reported [3]. Cork is a natural, renewable, and sustainable material with a variety of commercial applications; however, at this time, it was proposed as a promising material for monitoring devices due to its reactive chemical properties. The low-cost cork–graphite sensor was successfully used for determining caffeine in soft drinks and pharmaceutical formulations, highlighting the potential practice of these types of sensors for environmental purposes. Meanwhile, another novel electrochemical sensor based on homobifunctional tridentate disulfide Schiff base was reported in the field of environmental and health care methodologies for a selective and sensitive determination of toxic cations, such as Cr^3+^ [4]. It was found to be effective in its electrochemical response for Cr^3+^ in presence of other heavy metal cations, extending its technological advantages.

Reports regarding the recent advances in electrochemical processes for the destruction of pollutants were also pointed out for potential anodic electro-desulfurization [5], sequenced electrooxidation–plasma treatment [6], electrochemical chloride removal [7], and electrodisinfection [8]. By applying anodic potentials, the sulfur partition ratio was determined, allowing the proposal of an anodic electro-desulfurization mechanism, which will allow further advances on pyrometallurgy processes [5]. Meanwhile, important benefits could be achieved by using electrochemical oxidation as an alternative to removing azo dyes [6] and chloride [7] from different water matrices in order to promote advantages in the food processing industry and concrete construction sectors, respectively. In the former, the degradation of azo dye by sequential electrooxidation–plasma treatments, with boron-doped diamond and tungsten electrodes, was efficiently achieved via oxidizing species such as hydrogen peroxide and hydroxyl radicals, while the electrochemical chloride removal from reinforced concrete was proved to reduce the corrosion and passivation behaviors by an ultrasound-assisted method. 

A key role of diamond films as a potential electrocatalytic material in electrochemical technologies was presented when the deactivation of microorganisms and the destruction of micro-pollutants contained in synthetic urine wastewaters was demonstrated [8]. In this case, higher concentrations of active chlorine including hypochlorite ions and chloramines contributed to the effective inactivation of *E. coli* and the degradation of organic compounds in the synthetic urine wastewaters to mimic secondary treated sewage effluents. However, higher amounts of perchlorates were measured at the end of the electrolysis using this anode.

Finally, special attention was focused on the use of sequential processes considering that the main aspects that were related to chemical and technological advantages and disadvantages of electrochemical approaches [9]. Thus, an interesting report summarized and discussed the combination of anodic oxidation (AO) [10] and soil remediation technologies [11]. The decontamination of soil via ex situ and in situ AO for treating the effluents from soil remediation was the scope of this review. Moreover, the concept of soil washing, soil flushing, and electrokinetic as soil remediation techniques were briefly discussed, in addition to the importance of the electrocatalytic material on the AO process and the use of renewable energies [12].

Based on the wide gamut of subjects considered in the special issue, it should be of interest to a large number of diverse readers who are interested in both monitoring and environmental treatment technologies. In addition, this edition could be used for chemistry, electrochemistry, engineering, materials science, biotechnology, environmental engineering, and chemical engineering researchers and students focused on electrochemical advanced oxidation processes. We strongly believe that the future for electrochemical processes is bright in the forthcoming years to the benefit of our society. 
